# Up Front Unfolded Protein Response Combined with Early Protein Secretion Pathway Engineering in *Yarrowia lipolytica* to Attenuate ER Stress Caused by Enzyme Overproduction

**DOI:** 10.3390/ijms242216426

**Published:** 2023-11-17

**Authors:** Xingyu Zhu, Moying Li, Rui Zhu, Yu Xin, Zitao Guo, Zhenghua Gu, Liang Zhang, Zhongpeng Guo

**Affiliations:** 1 National Engineering Research Center for Cereal Fermentation and Food Biomanufacturing, Jiangnan University, Wuxi 214122, China; 17751494930@163.com (X.Z.); wuweisong2009@126.com (M.L.); zhuruiustc@163.com (R.Z.); yuxin@jiangnan.edu.cn (Y.X.); guzhenghua2011@163.com (Z.G.); zhangl@jiangnan.edu.cn (L.Z.); 2 Jiangsu Provincial Engineering Research Center for Bioactive Product Processing, Jiangnan University, 1800 Lihu Avenue, Wuxi 214122, China; 3 School of Food and Biological Engineering, Jiangsu University, Xuefu Road 301, Jingkou District, Zhenjiang 212013, China; guozt@ujs.edu.cn

**Keywords:** *Yarrowia lipolytica*, recombinant protein, ER stress, ER chaperones, translocation

## Abstract

Engineering the yeast *Yarrowia lipolytica* as an efficient host to produce recombinant proteins remains a longstanding goal for applied biocatalysis. During the protein overproduction, the accumulation of unfolded and misfolded proteins causes ER stress and cell dysfunction in *Y. lipolytica*. In this study, we evaluated the effects of several potential ER chaperones and translocation components on relieving ER stress by debottlenecking the protein synthetic machinery during the production of the endogenous lipase 2 and the *E. coli* β-galactosidase. Our results showed that improving the activities of the non-dominant translocation pathway (SRP-independent) boosted the production of the two proteins. While the impact of ER chaperones is protein dependent, the nucleotide exchange factor Sls1p for protein folding catalyst Kar2p is recognized as a common contributor enhancing the secretion of the two enzymes. With the identified protein translocation components and ER chaperones, we then exemplified how these components can act synergistically with Hac1p to enhance recombinant protein production and relieve the ER stress on cell growth. Specifically, the yeast overexpressing Sls1p and cytosolic heat shock protein Ssa8p and Ssb1p yielded a two-fold increase in Lip2p secretion compared with the control, while co-overexpressing Ssa6p, Ssb1p, Sls1p and Hac1p resulted in a 90% increase in extracellular β-galp activity. More importantly, the cells sustained a maximum specific growth rate (μ_max_) of 0.38 h^−1^ and a biomass yield of 0.95 g-DCW/g-glucose, only slightly lower than that was obtained by the wild type strain. This work demonstrated engineering ER chaperones and translocation as useful strategies to facilitate the development of *Y. lipolytica* as an efficient protein-manufacturing platform.

## 1. Introduction

Recombinant proteins (r-proteins) are valuable products broadly used in laboratory and industrial settings for various applications, ranging from biopharmaceuticals to industrial enzymes [1]. As a more sustainable and economic alternative to protein extraction from natural sources, microbial r-protein production represents a current multibillion-dollar market which is continuously expanding rapidly [1,2]. This stimulates an ever-greater need for innovative technologies for producing a high amount of high-quality r-proteins [3].

Currently, with advancements in emerging biotechnology tools, many genetically modified expression systems are available for commercial r-protein production, ranging from prokaryotes such as bacteria, to eukaryotes including yeasts, fungi and mammals, etc. [4]. Among them, yeasts are regarded as advantageous r-protein expressing systems as they are easy to culture and manipulate, and more importantly, are able to perform post-translational modifications [4].

In this regard, *Yarrowia lipolytica* stands out as an extraordinary protein expressing system for industrial applications. First of all, it has a “generally recognized as safe” (GRAS) status and there is a long history of usage of this yeast in food and pharmaceutical industries [5]. In addition, it is able to secrete large quantities of extracellular proteins [6] and it can be cultivated to a high density [7]. Moreover, it performs a wide range of posttranslational modifications (PTMs) [6,8]. Particularly, compared to other yeasts such as *Saccharomyces cerevisiae*, *Y. lipolytica* encounters reduced hyper-glycosylation issues, exhibiting glycosylation patterns closer to mammalian cells [1]. Over the past decades, various approaches have been implemented to improve heterologous protein production by *Y. lipolytica*, including optimizing cultivation conditions [9], engineering signal peptides [10], using high-strength promoters [11], increasing plasmid copy numbers [12], co-expressing secretory helpers [13] and decreasing intercellular and extracellular proteolysis [14], etc. Although more than 150 r-proteins can be expressed using *Y. lipolytica*, only 16% of them reached were able to produce at a bioreactor scale [1]. The engineering of *Y. lipolytica* as an efficient host for r-proteins production thus remains a longstanding challenge of applied biocatalysis.

**Figure 1:**
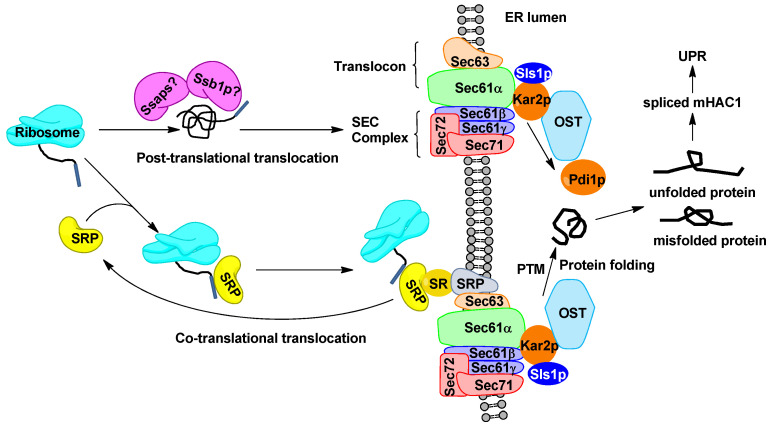
A schematic illustration of early secretory pathway and the spliced m*HAC1* mediated unfolded protein response (UPR) in *Y. lipolytica*. The predicted crucial components of translocation and ER folding chaperones are shown. Components with non-confirmed functions are indicated by a quotation mark. Detailed information about the key individual elements depicted in the figure can be found in Table S1 (Supplementary File S1). ? represents the predicted post-translational translocation components whose roles need to be confirmed. Notably, the co-translational translocation pathway is shown to be dominant and essential in *Y. lipolytica* [17].

In the early secretory pathway in yeasts, proteins targeting endoplasmic reticulum (ER) lumen may occur at two different processes, co- or post-translational translocations (Figure 1). The co-translational translocation is mediated by signal recognition particles (SRPs) which bind the signal peptide of the nascent secretory protein as soon as it emerges from the ribosome during translation. SRP causes a transient translation arrest and directs the ribosome-nascent chain complexes (RNCs) to the ER membrane-bound SRP receptor (SR) [15]. Once the RNC docked at the translocon, the SRP and SR dissociate, the synthesis of polypeptide proceeds as it passes through the Sec61 complex into the ER lumen [16]. The SRP of yeast generally consists a 7S RNA and six subunits (Sec65p, Srp14p, Srp21p, Srp54p, Srp68p and Srp72p). Among these subunits, Srp14p is essential for elongation arrest in translation by interacting with the RNA component, and Srp54p targets RNCs to ER via binding to the signal peptide of the nascent chain [17]. Alternatively, translocation may occur after translation in a SRP-independent manner (post-translational translocations) [18]. In this case, the newly synthesized proteins are stabilized by cytosolic chaperones of SSA subfamily of 70kDa heat shock proteins (Hsp70) and move towards ER by interacting directly with the Sec61 and Sec62/63 complex [19]. In this process, the ER chaperone Kar2p bounds to the unfolded protein and pulls the protein pass through the translocon [20]. Four SSA proteins sharing partially overlapping functions assist nascent proteins with folding and translocation. Among them, Ssa1p/2p are expressed constitutively, whereas Ssa3p/4p are expressed under induction in *S. cerevisiae* [21]. Thereafter, the nascent polypeptide is subjected to a series of modifications, including cleaving the N-terminal domain by a signal peptidase, introducing core glycosylation by oligosaccharyl transferase (OST), and forming disulfide bonds by Pdi1p (protein disulfide isomerase), leading to correct protein folding and mature structure [22].

Before reaching their destination, the secretory polypeptides are strictly examined by multiple quality-control points traverse across the secretory pathway. During the protein overproduction, the accumulation of unfolded or misfolded proteins may occur in the over-loaded ER, thus triggering the unfolded protein response (UPR) which protects the cells from ER stress or cellular dysfunction by debottlenecking the synthesis-folding-secretion pathway [23]. In the UPR, the endoribonuclease Ire1p signals the ER stress to remove an intron of 29 nt from the *HAC1* mRNA [24,25], generating spliced *HAC1* mRNA that can be translated into the active protein. Hac1p binds to the UPR elements (UPRE) and mediates the transcription of the genes involved in polypeptide formation, folding and maturation to restore cellular homeostasis [26].

In this context, engineering the protein folding and translocation has been reported as a useful strategy to improve protein secretion in different hosts. For instance, studies have shown that overexpressing SRP proteins such as Srp14p, the essential component involved in co-translational translocation, has increased the secretion of immunoglobulin (IgG) protein and Infliximab in Chinese hamster ovary cells (CHOs) [27]. Overexpressing Srp14p and Srp54p can enhance the secretion of heterologous proteins β-glucosidase, endoglucanase, and α-amylase in *S. cerevisiae* [28]. In addition, overexpressing Ssa1p, which plays an important role in post-translational translocation, can improve β-glucosidase secretion in *S. cerevisiae* and the granulocyte colony stimulating factor (G-CSF) in *Pichia pastoris* [28]. On the other hand, overexpressing key ER components assisting protein folding and/or translocation, such as the chaperone Kar2p and Pdi1p (disulfide isomerase), can enhance the production of a variety of proteins in CHOs [29], *Pichia pastoris* [30] and *S. cerevisiae* [28]. The above results indicate that the protein translocation and folding might be rate-limiting steps in protein secretion. Recently, the beneficial effects of overexpressing some of these components, including SSA5 and SSA8 on the secretion yield of YFP, has been demonstrated in *Y. lipolytica* for the first time [13]. However, more evidence is needed to confirm if these components are general ‘helper’ proteins or protein specific. In addition, the roles of other essential components, such as the Hsp70 homolog Ssbp, which binds directly to the ribosome and contacts a variety of newly synthesized polypeptide chains [31], and the ER-localized protein Sls1p, which interacts directly with Kar2p and is essential for protein translocation into the ER, may also play important roles in protein secretion [32]. Also, attempts have been made on enhancing the protein secretion and folding in eukaryotes by *HAC1* overexpression [23,33].

In this work, we have overexpressed several potential ER chaperones and translocation components and studied their effects on the production of an endogenous lipase (Lip2) and a heterologous β-galactosidase from *E. coli* in *Y. lipolytica*. Particularly, the effect of co-overexpression of Hac1p on attenuation of ER stress and enhancement of the capacity of cytosolic folding of the target r-proteins were studied. By comparing the expression level of these proteins, the essential components were identified and their roles in improving protein synthesis were deduced. Our work provides insight on the rational design of engineered strains of *Y. lipolytica* with enhanced protein secretion and folding capacity, which holds great potential for the industrial production of r-proteins.

## 2. Results and Discussion

### 2.1. Enzyme Overproduction Causes ER Stress and Triggers the Unfolded Protein Response (UPR)

In order to evaluate how protein over-expression may cause accumulation of unfolded or misfolded proteins that triggers ER stress in host cells, two enzymes, a native lipase 2 (Lip2p), and a heterologous β-galactosidase (β-galp), were overexpressed in *Y. lipolytica* using promoters at different strengths. Specifically, pTEF represents the construct the native *TEF* promoter. P4UASTEF and p8UASTEF represent the constructs expressing the genes under the hybrid *TEF* promoter, combined with four and eight tandem copies of UAS, respectively. 2×p8UASTEF represents the construct expressing two copies of the target gene under the hybrid *TEF* promoter combined with eight tandem copies of UAS.

**Figure 2:**
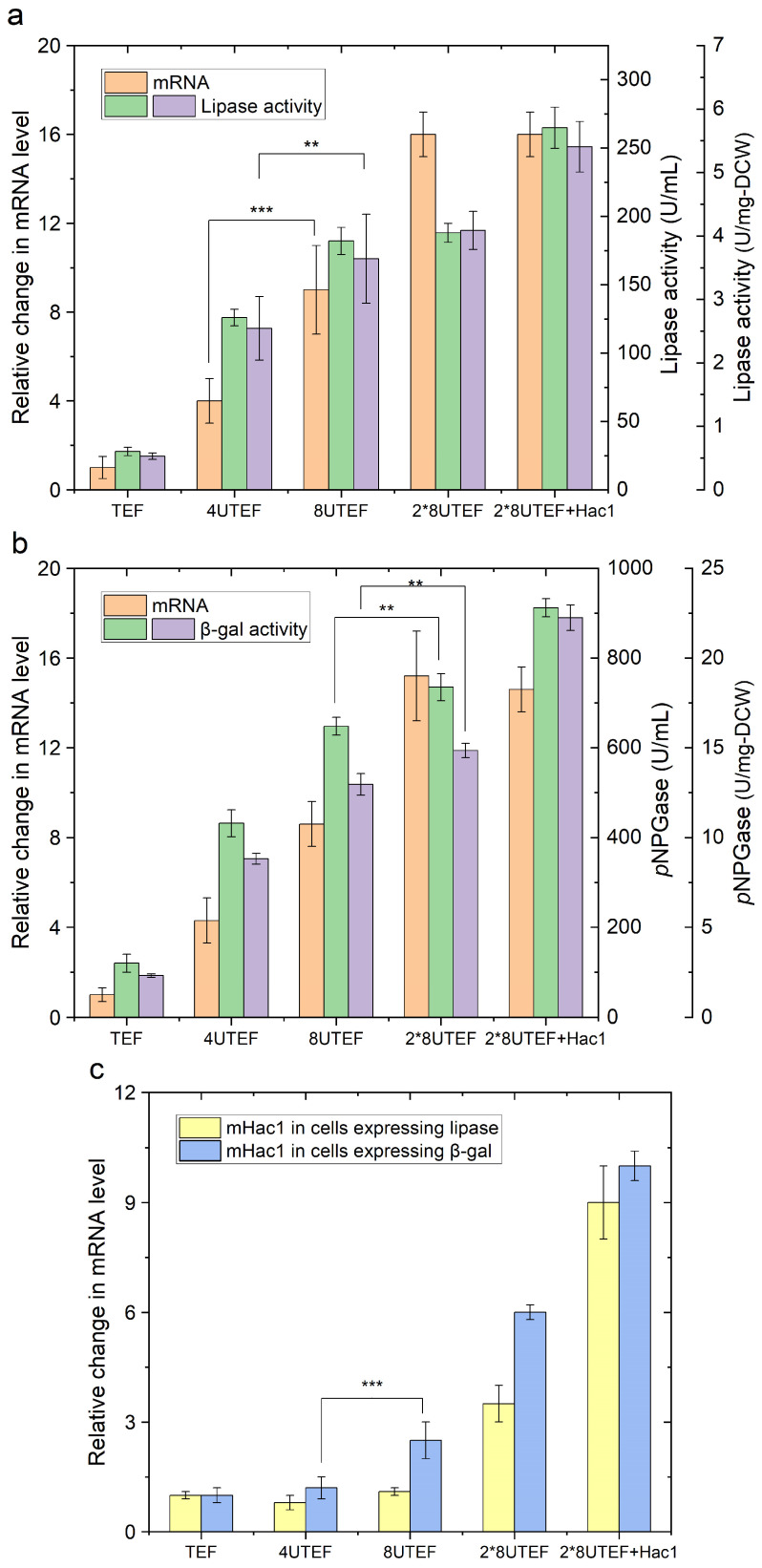
Comparison of the recombinant protein production and the relative cellular level of mRNA in *Y. lipolytica*. TEF, 4UTEF and 8UTEF represent the recombinant strains expressing the target gene under the native *TEF* promoter, hybrid *TEF* promoters combined with 4, and 8 tandem copies of UAS, respectively. 2×8UTEF represents the recombinant strain expressing two copies of the target gene under the hybrid *TEF* promoter combined with 8 tandem copies of UAS. 2×8UTEF + Hac1 represents the strain 2×8UTEF further overexpressing spliced m*HAC1*. (**a**) the mRNA level of *LIP2* and the corresponding extracellular lipase activity of *Y. lipolytica* expressing *LIP2* gene under the control of promoters with different strength; (**b**) the mRNA level of *lacZ* and the corresponding extracellular β-galactosidase activity of *Y. lipolytica* expressing *lacZ* gene under the control of promoters with different strength; (**c**) cellular mHAC1 level of *Y. lipolytica* expressing *LIP2* gene and *lacZ* gene under the control of promoters with different strength (** *p* value < 0.05, *** *p* value < 0.01, two-tailed Student’s *t*-tests).

The results showed that the expression level of both enzymes increased at the transcriptional level (reflected by mRNA) following the order: pTEF < p4UASTEF < p8UASTEF < 2×p8UASTEF (Figure 2). The elevated expression level by around five-fold using 4UASTEF, nine-fold using 8UASTEF, and sixteen-fold using two copies of 8UASTEF over pTEF was achieved. Interestingly, a different tendency was observed for protein production at the translational level (reflected by enzyme activity). Specifically, the lipase and β-galactosidase activities using 4UASTEF were elevated by 4.5-fold and 3.6-fold, respectively, compared with TEF (Figure 2a,b). Overexpressing lipase and β-galactosidase using 8UASTEF resulted in further improvement of enzyme production by 6.5-fold and 5.4-fold, respectively, over that of TEF. However, using two copies of 8UASTEF did not yield further improvement of enzyme activities compared with the use of single copy of 8UASTEF. Monitoring the level of spliced m*HAC1* revealed an increase in its transcripts by about 1.4 folds and 2.0 folds when 8UASTEF was used for the expression of Lip2p and β-galp, respectively, while a sudden increase of spliced m*HAC1* by above 9.0 folds was observed when 2×p8UASTEF was used for the expression of the two enzymes (Figure 2c).

Consistent with previous observations, these results indicated that the determinant of protein product level is not only at the transcriptional level, but also on translational level, more specifically, by the efficiency of protein synthesis machinery, and on the efficient secretion [34,35]. Obviously, the processing capacity of ER plays an essential role in protein production. In case of protein expression under super strong promoter 2×p8UASTEF, the abundancy of the transcripts did not necessarily mean hyper protein production as the production over-loaded the capacity of ER to handle polypeptides to form correctly folded proteins. Thus, an accumulation of unfolded or misfolded proteins occurred, which triggered UPR as indicated by a high level of spliced m*HAC1* as a hallmark. Strikingly, when the spliced m*HAC1* was co-overexpressed with the two enzymes under the control of 2×p8UASTEF, a further increase of Lip2p and β-galp by 9.4-fold and 7.6-fold, respectively, over *TEF* promoter was achieved (Figure 2). Therefore, *HAC1* overexpression enhanced secretion of the correctly folded target protein. This was probably achieved by relieving the UPR. These results were consistent with previous studies, which demonstrated that the capacity of yeast and fungal host cells for the secretion and folding of recombinant proteins was improved by *HAC1* overexpression [30,36].

### 2.2. Engineering Protein Translocation Improves Protein Production in Y. lipolytica

**Figure 3:**
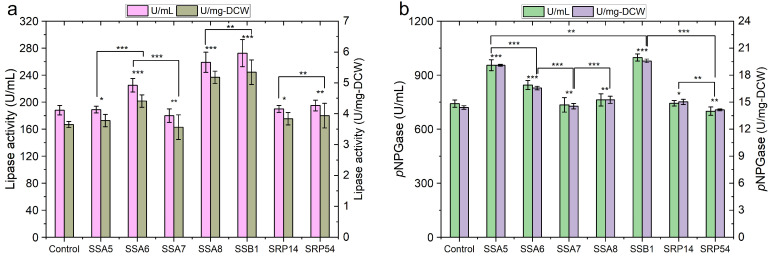
Effects of overexpressing key translocation components on protein production by recombinant *Y. lipolytica*. (**a**) lipase activity in culture supernatant of *Y. lipolytica* co-expressing Lip2p and Ssa5p, Ssa6p, Ssa7p, Ssa8p, Ssb1p, Srp12p or Srp54p, respectively; (**b**) β-galactosidase activity in culture supernatant of *Y. lipolytica* co-expressing β-galp and Ssa5p, Ssa6p, Ssa7p, Ssa8p, Ssb1p, Srp12p or Srp54p, respectively. The highest level of each protein was shown (typical after cultivated for 72 h on YTD). Data are presented as the mean value and standard deviation from at least three biological replicates (* *p* value > 0.05, ** *p* value < 0.05, *** *p* value < 0.01, two-tailed Student’s *t*-tests, the symbol alone without underneath indication bar represents the comparison was done with the control without overexpressing of the translocation components and ER chaperones.).

In the early secretory pathway, *Y. lipolytica* relies on two systems, co-translational translocation (SRP-dependent) and post-translational translocation, to target the nascent polypeptide to the ER lumen. To study the effects of engineering translocation system for r-protein production, we overexpressed several key translocation components including SSA subfamily (Ssa5p to Ssa8p), Ssb1p, Srp14p and Srp54p in *Y. lipolytica* expressing Lip2p and β-galp. Only the protein overexpression under 2*8UASTEF was studied since the cells suffered from a severe UPR. We first verified the expression of these r-proteins and investigated the physiological characteristics of the engineered *Y. lipolytica* (Supplementary File S1, Table S3). We confirmed that overexpressing these translocation components did not obviously influence the growth of the yeast cells. In addition, overexpression of the SSA subfamily and Hsp70 homolog improved the protein secretion (Figure 3). Generally speaking, the effect of Ssa7p overexpression on the secretion of active Lip2p and β-galp was not significant. Ssa6p overexpression increased the secretion of Lip2p by 20%, although to a less extent than Ssa8p, for which an above 38% increase in Lip2p production was achieved. In case of β-galp expression, the enhanced enzyme secretion was more pronounced for Ssa6p overexpression, for which an increase by 30% of β-galp activity was detected, while overexpression of Ssa5p led to a 15% increase in β-galp secretion. Remarkably, overexpressing Ssb1p in yeast significantly improved the active r-protein secretion, as more than a 45% increase in extracellular activities was registered for both enzymes (Figure 3). 

In post-translational translocation, cytosolic Hsp70s cochaperones, such as SSA subfamily and Hsp70 homolog Ssbp, bind newly synthesized proteins to stabilize their folding competent status, prevent the aggregation and assist in protein folding [37], which may have contributed to higher protein expression. The fact that the four Ssap orthologs of *Y. lipolytica* showed protein-dependent effects on their expression indicated the individual representatives of Ssap family possess redundant but distinct functionality, as has been suggested in previous study [38], even though the four Ssaps share high sequence similarity. Taken together, these results illustrate that increasing the activities of the non-predominant pathway of translocation may enhance its role as an alternative route targeting the nascent polypeptide to the ER lumen in yeast.

In *Y. lipolytica*, approximately 75% of the translocation pores Sec61p were linked to ribosomes and ER resident proteins Sls1p-Kar2p complexes [17], and disruption of the genes encoding SRP is lethal for the cells [39]. These data clearly demonstrated the dominant and crucial role of the SRP-dependent translocation pathway in this yeast. Surprisingly, out attempt to increase r-proteins production by overexpressing core co-translocation components Srp14p and Srp54p was unsuccessful (Figure 3). By contrast, *S. cerevisiae* shows a high preference towards post-translational translocation (SRP-independent), and co-translational translocation (SRP-dependent) is not essential for the cell survival [40], while the over-expression of Srp14p and Srp54p enhanced the secretion of heterologous proteins [28]. In case of *Y. lipolytica*, further experiments are needed to draw conclusion on the impact of Srp14p and Srp54p on r-protein secretion. First of all, the functional over-expression of Srp14p and Srp54p under our conditions remained to be confirmed. In addition, the possible protein-dependent effect of Srp14p and Srp54p needs to be studied.

### 2.3. Engineering Protein-Folding Machinery Improves Protein Production in Y. lipolytica

**Figure 4:**
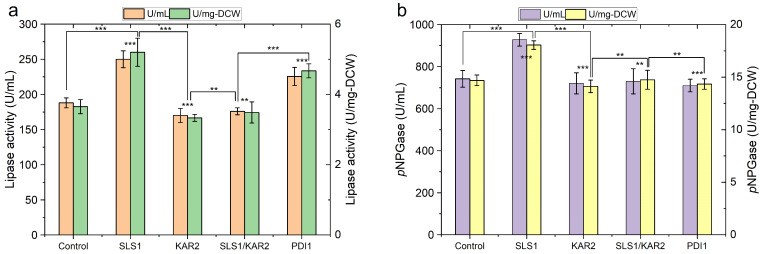
Effects of overexpressing ER chaperon es on protein production by recombinant *Y. lipolytica*. (**a**) lipase activity in culture supernatant of *Y. lipolytica* co-expressing Lip2p and Sls1p, Kar2p, Sls1p/Kar2p or Pdi1p, respectively; (**b**) β-galactosidase activity in culture supernatant of *Y. lipolytica* co-expressing β-galp and Sls1p, Kar2p, Sls1p/Kar2p or Pdi1p, respectively. The highest level of each protein was shown (typical after cultivated for 72 h on YTD). Data are presented as the mean value and standard deviation from at least three biological replicates (** *p* value < 0.05, *** *p* value < 0.01, two-tailed Student’s *t*-tests, the symbol alone without underneath indication bar represents the comparison was done with the control without overexpressing of the translocation components and ER chaperones.).

Following the process of translocation, the step of routing nascent polypeptide to ER lumen is mediated by Kar2p and Sec63p, which assist initial folding and the pore gating in *Y. lipolytica* [20]. We overexpressed the molecular chaperone Kar2p and its partner protein Sls1p, as well as disulfide isomerase Pdi1p to study the effects of increasing the folding capacity of *Y. lipolytica* on r-protein production. The Lip2p-producing strain overexpressing Sls1p showed a 33% increase in extracellular activity. Similarly, the secretion yield of β-galp was improved by 25% by overexpressing Sls1p in *Y. lipolytica*, whereas overexpressing Pdi1p showed no effect on β-galp production (Figure 4). On the contrary, Pdi1p overexpression enhanced Lip2p production by 20% compared to the control; overexpressing Kar2p or co-overexpressing Sls1p and Kar2p did not obviously change the production yield of the two r-proteins. 

Enhancing protein folding capacity by overexpressing Kar2p and Pdi1p have been shown to be useful strategy to improve r-protein production in *S. cerevisiae* [28]. However, our results revealed that their effects were protein dependent, and overexpressing Kar2p and Pdi1p may even impair the secretion of some proteins, such as β-galp.

In contrast to a minor role of its homolog Sil1p in *S. cerevisiae* [22], Sls1p is the key nucleotide exchange factor (NEF) in *Y. lipolytica* modulating ATPase activity of Kar2p by facilitating the exchange of ADP with ATP, mediating the interaction of the two crucial proteins Kar2p and Sec63p in co-translational translocation [17]. While deletion of Sil1p barely influences the translocation in *S. cerevisiae*, Sls1p deletion showed a strong impact on translocation of nascent secretory proteins in *Y. lipolytica* [17]. Apart from its crucial role in protein co-translational translocation, Sls1p is also involved in sensing and activating an unfolded protein response [17,20,25], all of which may have contributed to the increased level of r-protein secretion upon Sls1p’s overexpression.

The chaperone PDI1p catalyses disulfide bond formation, rearranges incorrect disulfides, reduces non-productive aggregation and acts together with protein folding catalyst by forming complexes to fold nascent proteins as in the ER [41]. For the two r-proteins expressed here, as expected, no disulfide bonds were predicted in β-galp originated from *E. coli* using online server DISULFIND (http://disulfind.dsi.unifi.it/.) accessed on 20 December 2022 [42]. By contrast, structure analysis showed Lip2p is stabilised by four disulfide bonds [43], which may explain the contribution of Pdi1p overexpression in enhancing the production of this protein. Our results reveal the importance of choosing the right strategies in engineering folding machinery to improve r-protein production.

### 2.4. The Impact of Engineering Protein Translocation and Folding on R-Protein Production in Y. lipolytica

After identification of the components involved in the translocation of nascent proteins and protein folding in the ER lumen, whose overexpression has enhanced the r-protein production, we studied the synergetic effects of the combination of these components on protein secretion.

**Figure 5:**
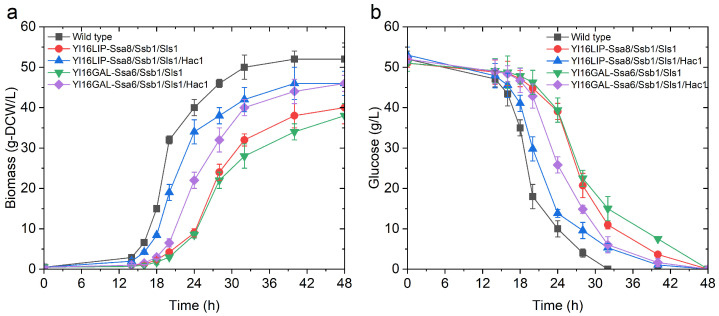
Comparison of the (**a**) growth and (**b**) glucose consumption of *Y. lipolytica* wildtype and the recombinant strains Yl16LIP-Ssa8/Ssb1/Sls1, Yl16LIP-Ssa8/Ssb1/Sls1/Hac1, Yl16GAL-Ssa6/Ssb1/Sls1 and Yl16GAL-Ssa6/Ssb1/Sls1/Hac1 during aerobic culture in YTD. Please note that only the growth profiles of the most relevant and the representative strains demonstrated significant difference from the wildtype were shown.

**Figure 6:**
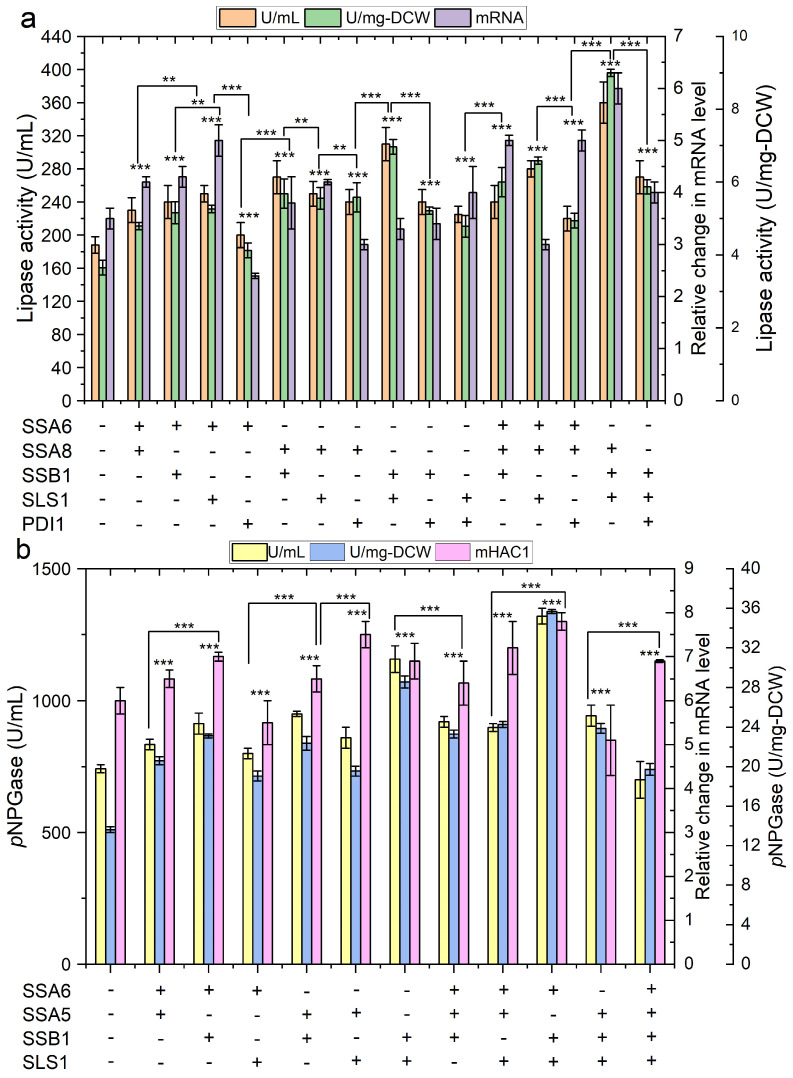
The effects of combination of protein translocation components and ER chaperones on protein production by recombinant *Y. lipolytica* and the detection of ER stress. (**a**) the activity of Lip2p in culture supernatant and the measurement of cellar mRNA level of *HAC1*; (**b**) the β-galactosidase activity in culture supernatant and the measurement of cellar mRNA level of *HAC1*. The highest level of each protein was shown (typical after cultivated for 72 h on YTD). “+” and “-” indicate the corresponding gene was or was not overexpressed respectively. Data are presented as the mean value and standard deviation from at least three biological replicates (** *p* value < 0.05, *** *p* value < 0.01, two-tailed Student’s *t*-tests, the symbol alone without underneath indication bar represents the comparison was done with the control without overexpressing of the translocation components and ER chaperones.).

Generally speaking, increasing the r-protein production inevitably reduced the specific growth rate of the above strains and the final biomass yield (Supplementary File S1, Table S3, Figure 5), which have been ascribed to the increased energy expenditure and metabolic burden for protein production in previous reports [34,35]. Notably, although the synergetic effects benefit Lip2p production were registered for all the possible combinations of Ssa6p, Ssa8p, Ssb1p, Sls1p and Pdi1p, co-overexpression of Ssa8p, Ssb1p and Sls1p was shown to be the best, which yielded two-folds Lip2p production over the control (Figure 6a). Co-overexpressing either two or three proteins among Ssa5p, Ssa6p, Ssb1p and Sls1p improved the production of β-galp, while the boosting effect was more pronounced for Ssa6p, Ssb1p and Sls1p co-overexpression (90% increase in extracellular β-galp activity) (Figure 6b). However, a combination of more than three of the key components of protein translocation and folding pathway was harmful for the production of both proteins. The elevated expression of *HAC1* at a transcriptional level indicated the above cells suffered from severe ER stress. As a result, the µmax of the best protein production strain Yl16LIP-Ssa8/Ssb1/Sls1 (lipase 2 overexpressing strain) and Yl16GAL-Ssa6/Ssb1/Sls1 (β-galp overexpressing strain) was decreased by 30% and 42%, respectively, compared with the control (Supplementary File S1, Table S3). Interestingly, overexpressing Hac1p in Yl16LIP-Ssa8/Ssb1/Sls1 and Yl16GAL-Ssa6/Ssb1/Sls1 not only improved the secretion yield of the target proteins further, but also relived the metabolic burden of r-protein synthesis on the biomass formation, as elevated growth rates by up to 88% of the wild type were observed for both strains (Figure 5 and Figure 6; Supplementary File S1, Table S3). Noteworthy, the hyper-productive strains exhibited a filamentous growth instead of ovoid form, which indicates the cells were under severe stress caused from metabolic burden due to recombinant protein production [44]. The dimorphism of this yeast may result from the remodelling of cellular membranes and cell wall structures, as well as potent vesicular transportation [22]. As demonstrated in previous study, the ovoid cells are generally more efficient r-protein producers than filamentous cells [44]. Further study is needed to identify the molecular mechanism involved in driving dimorphic transition and use this knowledge to guide the engineering of *Y. lipolytica* with a high capacity in heterologous protein secretion. 

Taken together, these results imply that the protein production may be limited by the early secretory pathways and folding processes, and enhancing the translocation and protein folding capacities of the yeast cells may benefit the r-protein secretion.

## 3. Materials and Methods

### 3.1. Strains, Reagents and Culture Media

The microbial strains used in this work are summarized in Table 1. All reagents were ordered from Sigma-Aldrich unless otherwise stated (Shanghai, China). DNA polymerase and restriction enzymes were purchased from Vazyme (Vazyme Biotech, Nanjing, China). For *Y. lipolytica* cultivation, a Yeast Extract Peptone Dextrose (YPD) medium containing 10 g/L yeast extract, 10 g/L peptone and 10 g/L glucose was used. Transformant selection was performed on a solid Yeast Nitrogen Base (YNB) medium, composed of 1.7 g/L YNB, 10 g/L glucose, 5 g/L ammonium chloride. Leucine or uracil was supplemented into the media at the concentration of 440 mg/L according to the auxotrophic marker requirement. For solid media, 1.5% agar was added. R-proteins were produced in a Yeast Extract Tryptone Dextrose (YTD) medium containing 10 g/L yeast extract, 20 g/L tryptone and 50 g/L glucose, buffered by 100 mM phosphate at pH 6.8 in shake flasks.

### 3.2. Plasmid and Strain Construction

The gene targets and their putative functions, as well as primers used for gene amplification, are summarized in Table S1. The plasmids constructed are listed in Table S2 (Supplementary File S1). First of all, the plasmids pYL1/pYU1, pYL4/pYU4 and pYL8/pYU8, derived from the plasmid pYLXP [45], were constructed. The plasmid pYL1 contains a *LEU2* selection marker flanking with a *loxP* site and a 500-bp sequence on each end, one of which is homologous to the upstream and the other to the downstream of *URA3* gene. Similarly, pYU1 contains a *URA3* selection marker flanking with a *loxP* site and a 500-bp sequence on each end, one of which is homologous to the upstream and the other to the downstream of *LUE2* gene. Replacing the *TEF* promoters of the above plasmids by the 4UASTef (combination of 4 tandem copies of upstream activation sequences (UAS) with the core *TEF* promoter element) and 8UASTef promoter, yields the plasmids pYL4/pYU4 and pYL8/pYU8 [8].

The genes encoding putative Hac1p, Kar2p, Sls1p, Srp14p, Srp54p, Ssa5p, Ssa6p, Ssa7p, Ssa8p, Ssb1p and Pdi1p in *Y. lipolytia* were identified by multi-sequence alignment with their homologs in *S. cerevisiae*. After that, these genes were amplified from the gDNA of *Y. lipolytica* using Phanta Flash Master Mix (Dye Plus) (Vazyme Biotech, Nanjing, China) with primers listed in Table S1 (Supplementary File S1). The genes obtained were digested with *Xba*I and *Spe*I and cloned into the corresponding sites of plasmid pYL1 or pYU1 under the control of *TEF*. To express the spliced m*HAC1*, the plasmid pYL-spliced Hac1 was amplified by reverse PCR using primers rPCR-HACf/rPCR-HACr, followed by homologous recombination. This allows the removal of an intron of 29 nt (cagtgatgactgtcgcaactactgaccag) from the *HAC1* gene.

The gene *LIP2*, encoding the triacylglycerol lipase 2, was amplified from the gDNA of *Y. lipolytica* and cloned into the *Xba*I/*Spe*I sites of the plasmids pYU1, pYU4 and pYU8, to generate plasmid pYU1-LIP2, pYU4-LIP2 and pYU8-LIP2, respectively. The gene *lacZ* (GenBank accession number: NP_414878.1), encoding β-galactosidase in *E. coli* fused with the signal peptide of Lip2, was codon optimized and synthesized, and directly cloned into the plasmids pYU1, pYU4 and pYU8 to generate the plasmids pYU1-lacZ, pYU4-lacZ and pYU8- lacZ, respectively. After construction, the correct sequence of the recombinant plasmid was verified by DNA sequencing (Tianlin Biotech, Wuxi, China).

For yeast transformation, the plasmids were linearized and introduced into *Y. lipolytica* po1f using the Zymo Research Frozen-EZ Yeast Transformation II Kit (Irvine, CA, USA). Transformant selection was performed on a YNB plate according to the auxotrophic genotype. The reuse of the selection markers was achieved by introducing the *loxP*-*Cre* recombination system into the transformants during multistep incorporation of the target genes [45]. The success in incorporating multiple genes into the *Y. lipolytica* genome was verified by PCR using gene specific primers. Subsequently, 10 transformants of each construct were grown in liquid YPD media, and the recombinant strains that showed the representative phenotype and produced the representative level of r-proteins were selected for further investigation.

### 3.3. Recombinant Protein Production

The yeast strains were first cultivated in YPD media overnight. Then, this preculture was used to inoculate 100 mL YTD media in a 1 L Erlenmeyer flask at an initial OD value of 0.5. The cultures were incubated at 28 °C under continuous shaking at 150 rpm for 5 days. Samples were taken regularly to monitor the growth, enzyme production, glucose consumption and biomass formation.

### 3.4. Gene Expression Analysis at Transcriptional Level

For gene expression analysis, mRNA was extracted using the Simple P Total RNA Extraction Kit (BSC52S1; BIOER, Hangzhou, China). cDNA was synthesized and amplified using the Prime Script RT Kit (TaKaRa Bio Inc., Kusatsu, Shiga, Japan). *ACT1* gene (YALI0_D08272g), encoding actin in *Y. lipolytica*, was used as the reference. *LIP2*, *LacZ*, *ACT1* and *HAC1* were amplified using the primer pairs qlip2-f/qlip2-r, qlacZ-f/qlacZ-r, qact1-f/qact1-r and qhac1-f/qhac1-r, respectively. RT-qPCR was conducted using the Universal YSBR qPCR Master Mix (Vazyme Biotech, Nanjing, China) in a qTOWER2.0 thermal cycler (Analytik Jena, Jena, Germany). Relative gene expression level is shown as fold change (FC) over the reference gene. 

### 3.5. Measurement of Enzyme Activity

Samples of culture broth were subjected to centrifugation at 8000× *g* for 5 min at 4 °C, thus allowing the isolation of the culture supernatant. β-Galactosidase activity was measured by quantifying the release of *p*NP (*p*-nitrophenol) from *p*-nitrophenyl-β-d-galactopyranoside (*p*NPG), as described previously [46]. One unit of activity was defined as the enzyme quantity needed to release 1 μmol *p*NP per min. The measurement of lipase activity was conducted by monitoring the release of p-nitrophenol from *p*-nitrophenyl butyrate (*p*NPB), as described previously [47]. The protein concentration was determined using a BCA (Bicinchoninic Acid) Protein Assay Kit (Merck, Rahway, NJ, USA), according to the manufacturer’s instruction, using bovine serum albumin as the standard [48]. All the activity assays were performed at least in triplicate.

### 3.6. Analysis of Glucose Consumption and Biomass Formation

The glucose concentration in culture supernatant was analysed using HPLC equipped with an Aminex HPX87-H column (Bio-Rad, Feldkirchen, Germany) and a Shodex RI-101 refractive index detector (Dionex, Sunnyvale, CA, USA). During the analysis, the column was kept at 50 °C and 5 mM H_2_SO_4_ was used as the mobile phase at a flowing rate of 0.6 mL/min. 

To quantify the dry cell weight, the culture broth was filtered using a pre-weighed PES membrane (0.45 μm; Sartorius Biolab, Berlin, Germany), and then, the biomass retained on the filter was washed, dried and weighed.

### 3.7. Statistical Analysis

The results were analysed by two-tailed Student’s *t*-tests, wherein *p* values ≤ 0.05 were considered statistically significant.

## 4. Conclusions

In the present work, we identified several potential ER chaperones and translocation components. We demonstrated that improving the activities of the non-dominant translocation pathway (SRP-independent) boosted the production of the two proteins. In addition, we found that the impact of ER chaperones is protein dependent; the nucleotide exchange factor Sls1p for the protein-folding catalyst Kar2p is recognized as a common contributor enhancing the secretion of the two recombinant proteins, Lip2p and β-galp. While co-overexpression of Ssa8p, Ssb1p and Sls1p was shown to be the best, which yielded a two-fold increase in Lip2p production, the boosting effect was more pronounced for Ssa6p, Ssb1p and Sls1p co-overexpression for β-galp production. Further enhancing Hac1p expression in the above strains did not only improve the enzyme secretion, but also largely restored the growth of the strains up to 88% of the wild type. This work proved that engineering ER chaperones and translocation are useful strategies to facilitate the development of *Y. lipolytica* as an efficient protein-manufacturing platform.

## Figures and Tables

**Table 1:** Microbial strains used in the present study.

Strains	Relevant Genotype	Source of Reference
*E. coli* DH5	Φ80dlacZΔm15, *recA1*, *endA1*, *gyrA96*, *thi-1*, *hsdR17* (rk^−^, mk^+^), *supE44*, *relA1*, *deoR*, Δ(*lacZYA*-argF) U169	Invitrogen
*Y. lipolytica* Po1f	*MatA*, *leu2-270*, *ura3-302*, *xpr2-322 axp1*	[14]
YlLIP	Po1f; *pTEF-LIP2-URA3*, *LEU2*	This investigation
Yl4LIP	Po1f; *p4UASTEF-LIP2-URA3*, *LEU2*	This investigation
Yl8LIP	Po1f; *p8UASTEF-LIP2-URA3*, *LEU2*	This investigation
Yl16LIP	Po1f; *p8UASTEF-LIP2-URA3*, *p8UASTEF-LIP2-LEU2*	This investigation
Yl16LIP-Hac1 (Kar2, Sls, Sp14, Sp54, Ss5, Ss6, Ss7, Ss8, Sb or Pd)	Yl16LIP; *pTEF*-*HAC1* (or *KAR2*, *SLS1*, *SRP14*, *SRP54*, *SSA5*, *SSA6*, *SSA7*, *SSA8*, *SSB1*, *PDI1*)	This investigation
Yl16LIP-Ssa6/Ssa8	Yl16LIP; *pTEF-SSA6*, *pTEF-SSA8*	This investigation
Yl16LIP-Ssa6/Ssb1	Yl16LIP; *pTEF-SSA6*, *pTEF-SSB1*	This investigation
Yl16LIP-Ssa6/Sls1	Yl16LIP; *pTEF-SSA6*, *pTEF-SLS1*	This investigation
Yl16LIP-Ssa6/Pdi1	Yl16LIP; *pTEF-SSA6*, *pTEF-PDI1*	This investigation
Yl16LIP-Ssa8/Ssb1	Yl16LIP; *pTEF-SSA8*, *pTEF-SSB1*	This investigation
Yl16LIP-Ssa8/Sls1	Yl16LIP; *pTEF-SSA8*, *pTEF-SLS1*	This investigation
Yl16LIP-Ssa8/Pdi1	Yl16LIP; *pTEF-SSA8*, *pTEF-PDI1*	This investigation
Yl16LIP-Ssb1/Sls1	Yl16LIP; *pTEF-SSB1*, *pTEF-SLS1*	This investigation
Yl16LIP-Ssb1/Pdi1	Yl16LIP; *pTEF-SSB1*, *pTEF-PDI1*	This investigation
Yl16LIP-Sls1/Pdi1	Yl16LIP; *pTEF-SLS1*, *pTEF-PDI1*	This investigation
Yl16LIP-Ssa6/Ssa8/Ssb1	Yl16LIP; *pTEF-SSA6*, *pTEF-SSA8*, *pTEF-SSB1*	This investigation
Yl16LIP-Ssa6/Ssa8/Sls1	Yl16LIP; *pTEF-SSA6*, *pTEF-SSA8*, *pTEF-SLS1*	This investigation
Yl16LIP-Ssa6/Ssa8/Pdi1	Yl16LIP; *pTEF-SSA6*, *pTEF-SSA8*, *pTEF-PDI1*	This investigation
Yl16LIP-Ssa6/Ssb1/Sls1	Yl16LIP; *pTEF-SSA6*, *pTEF-SSB1*, *pTEF-SLS1*	This investigation
Yl16LIP-Ssa8/Ssb1/Sls1	Yl16LIP; *pTEF-SSA8*, *pTEF-SSB1*, *pTEF-SLS1*	This investigation
Yl16LIP-Ssb1/Sls1/Pdi1	Yl16LIP; *pTEF-SSB1*, *pTEF-SLS1*, *pTEF-PDI1*	This investigation
Yl16LIP-Ssa8/Ssb1/Sls1/Hac1	Yl16LIP; *pTEF-SSA8*, *pTEF-SSB1*, *pTEF-SLS1*, *pTEF*-spliced *HAC1*	This investigation
YlGAL	Po1f; *pTEF-gal-URA3*, *LEU2*	This investigation
Yl4GAL	Po1f; *p4UASTEF-gal-URA3*, *LEU2*	This investigation
Yl8GAL	Po1f; *p8UASTEF-gal-URA3*, *LEU2*	This investigation
Yl16GAL	Po1f; *p8UASTEF-gal-URA3*, *p8UASTEF-gal-LEU2*	This investigation
Yl16GAL-Hac1 (Kar2, Sls, Sp14, Sp54, Ss5, Ss6, Ss7, Ss8, Sb or Pd)	Yl16GAL; *pTEF*-spliced *HAC1* (or *KAR2*, *SLS1*, *SRP14*, *SRP54*, *SSA5*, *SSA6*, *SSA7*, *SSA8*, *SSB1, PDI1*)	This investigation
Yl16GAL-Ssa5/Ssa6	Yl16GAL; *pTEF-SSA5*, *pTEF-SSA6*	This investigation
Yl16GAL-Ssa5/Ssb1	Yl16GAL; *pTEF-SSA5*, *pTEF-SSB1*	This investigation
Yl16GAL-Ssa5/Sls1	Yl16GAL; *pTEF-SSA5*, *pTEF-SLS1*	This investigation
Yl16GAL-Ssa6/Ssb1	Yl16GAL; *pTEF-SSA6*, *pTEF-SSB1*	This investigation
Yl16GAL-Ssa6/Sls1	Yl16GAL; *pTEF-SSA6*, *pTEF-SLS1*	This investigation
Yl16GAL-Ssb1/Sls1	Yl16GAL; *pTEF-SSB1*, *pTEF-SLS1*	This investigation
Yl16GAL-Ssa5/Ssa6/Ssb1	Yl16GAL; *pTEF-SSA5*, *pTEF-SSA6*, *pTEF-SSB1*	This investigation
Yl16GAL-Ssa5/Ssa6/Sls1	Yl16GAL; *pTEF-SSA5*, *pTEF-SSA6*, *pTEF-SLS1*	This investigation
Yl16GAL-Ssa6/Ssb1/Sls1	Yl16GAL; *pTEF-SSA6*, *pTEF-SSB1*, *pTEF-SLS1*	This investigation
Yl16GAL-Ssa5/Ssa6/Ssb1/Sls1	Yl16GAL; *pTEF-SSA5*, *pTEF-SSA6*, *pTEF-SSB1*, *pTEF-SLS1*	This investigation
Yl16GAL-Ssa6/Ssb1/Sls1/Hac1	Yl16GAL; *pTEF-SSA6*, *pTEF-SSB1*, *pTEF-SLS1*, *pTEF*-spliced *HAC1*	This investigation

## Data Availability

All data generated or analyzed in the present study are included in this published article and a supporting material “Supplementary File S1”.

## Supplementary Materials

The supporting information can be downloaded at: https://www.mdpi.com/article/10.3390/ijms242216426/s1.
