# Supplementary material for: Up Front Unfolded Protein Response Combined with Early Protein Secretion Pathway Engineering in Yarrowia lipolytica to Attenuate ER Stress Caused by Enzyme Overproduction
Source: Int J Mol Sci. 2023 Nov 17;24(22):16426. doi: 10.3390/ijms242216426 (PMC10670989; doi:10.3390/ijms242216426)
Supplement: Supplementary file 1 [file ijms-24-16426-s001.zip › ijms-2655198-supplementary.pdf]

Plasmid and strain construction

The gene targets and their putative functions as well as primers used for gene amplification are summarized in Table S1. The plasmids constructed are listed in Table S2 (Supplementary File S1). First of all, the plasmids pYL1/pYU1, pYL4/pYU4 and pYL8/pYU8, derived from the plasmid pYLXP, were constructed. The plasmid pYL1 contains a *LEU2* selection marker flanking with a *loxP* site and a 500-bp sequence on each end, one of which is homologous to the upstream and the other to the downstream of *URA3* gene. Similarly, pYU1 contains a *URA3* selection marker flanking with a *loxP* site and a 500-bp sequence on each end, one of which is homologous to the upstream and the other to the downstream of *LUE2* gene. Replacing the *TEF* promoters of the above plasmids by 4UASTef (combination of 4 tandem copies of upstream activation sequences (UAS) with the core *TEF* promoter element) and 8UASTef promoter yield the plasmids pYL4/pYU4 and pYL8/pYU8. The genes encoding putative Hac1p, Kar2p, Sls1p, Srp14p, Srp54p, Ssa5p, Ssa6p, Ssa7p, Ssa8p, Ssb1p and Pdi1p in *Y. lipolytia*, were identified by multi-sequence alignment with their homologs in *S. cerevisiae*.

After that, these genes were amplified from the gDNA of *Y. lipolytica* using Phanta Flash Master Mix (Dye Plus) (Vazyme Biotech, Nanjing, China) with primers listed in Table S1 (Supplementary File S1). The genes obtained were digested with *Xba*I and *Spe*I and cloned into the corresponding sites of plasmid pYL1 or pYU1 under the control of *TEF*. To expressed the spliced m*HAC1*, the plasmid pYL-spliced Hac1 was amplified by reverse PCR using primers rPCR-HACf/rPCR-HACr followed by homologous recombination. This allows the removal of an intron of 29 nt (cagtgatgactgtcgcaactactgaccag) from the *HAC1* gene.

The gene *LIP2*, encoding the triacylglycerol lipase 2, was amplified from the gDNA of *Y. lipolytica* and cloned into the *Xba*I/*Spe*I sites of the plasmids pYU1, pYU4 and pYU8, to generate plasmid pYU1-LIP2, pYU4-LIP2 and pYU8-LIP2, respectively. The gene *lacZ* (GenBank accession number: NP_414878.1), encoding β-galactosidase in *E. coli* fused with the signal peptide of Lip2, was codon optimized and synthesized, and directly cloned into the plasmids pYU1, pYU4 and pYU8, to generate the plasmids pYU1-lacZ, pYU4-lacZ and pYU8- lacZ, respectively. After construction, the correct sequence of the recombinant plasmid was verified by DNA sequencing (Tianlin Biotech, Wuxi, China).

Gene expression analysis at transcriptional level

For gene expression analysis, mRNA was extracted using the Simple P Total RNA Extraction Kit (BSC52S1; BIOER, Hangzhou, China). cDNA was synthesized and amplified using the Prime Script RT Kit (TaKaRa Bio Inc., Kusatsu, Shiga, Japan). *ACT1* gene (YALI0_D08272g), encoding actin in *Y. lipolytica*, was used as the reference. *LIP2*, *LacZ, ACT1* and *HAC1* were amplified using the primer pairs qlip2-f/qlip2-r, qlacZ-f/qlacZ-r, qact1-f/qact1-r and qhac1-f/qhac1-r, respectively. RT-qPCR was conducted using the Universal YSBR qPCR Master Mix (Vazyme Biotech, Nanjing, China) in a qTOWER2.0 thermal cycler (Analytik Jena, Jena, Germany). Relative gene expression level is shown as fold change (FC) over the reference gene.

Table S1 Sequences of the oligonucleotide primers used in this study

Gene target	Locus	Putative function	Primer names	Sequence (5'-3')*
HAC1	YALI0_B12716g	transcription factor singleton	HACf	CGCTCTAGACACAATGTCTATCAAGCGAGAAGAGTCCTTTAC
			HACr	GGCACTAGTCTAGATCAGCAATAAAGTCGTGCTGG
Spliced HAC1	YALI0_B12716g	transcription factor singleton	rPCR-HACf	ACCGGGCGCGTCATCCAGCAGCGTCGGCACAAGATTTCATTTTCATCAAGG
			rPCR-HACr	GATGACGCGCCCGGTCCACAAGATAA
KAR2	YALI0_E13706g	molecular chaperone in ER translocation of secretory proteins	KARf	CGCTCTAGACACAATGAAGTTCTCTATGCCTTCGTGG
			KARr	GGCACTAGTTTAAAGCTCATCGTGGAAAGGAG
SLS1	YALI0_E32703g	Nucleotide exchange factor for Kar2p	SLSf	CGCTCTAGACACAATGAAGTTCAGCAAGACTCTACTACTGGC
			SLSr	GGCACTAGTTTAAAGCTCATCACGAGCGTCGT
SRP14	YALI0_F24013g	signal recognition particle subunit	SRP14f	CGCTCTAGACACAATGTCGAGACTGACCAACACAGAG
			SRP14r	GGCACTAGTCTACTTTCGCTTTCGCTTCTTGT
SRP54	YALI0_F03839g	Signal recognition particle 54 kDa protein	SRP54f	CGCACTAGTCACAATGGTTCTTGAAGACCTCGGAA
			SRP54r	GGCACTAGTTTAGATGCCGAGACCCATACTTC
SSA5	YALI0_F25289g	cytosolic heat shock protein of HSP70 family	SSA5f	CGCTCTAGACACAATGCCTAACACATCTGTAGGAATTGACCT
			SSA5r	GGCACTAGTTTAGTCGACCTCCTCGACAGTGGG
SSA6	YALI0_D08184g	cytosolic heat shock protein of HSP70 family	SSA6f	CGCTCTAGACACAATGTCCAAAGCAGTAGGAATTGAT
			SSA6r	GGCACTAGTTTAGTCAACCTCCTCAACGGTG
SSA7	YALI0_E35046g	cytosolic heat shock protein of HSP70 family	SSA7f	CGCTCTAGACACAATGTCTAAAGCAGTAGGAATCGATCTTG
			SSA7r	GGCACTAGTTTAGTCAACCTCCTCAACAGTGGG
SSA8	YALI0_D22352g	cytosolic heat shock protein of HSP70 family	SSA8f	CGCTCTAGACACAATGTCTAAAGCCGTCGGAATCGATC
			SSA8r	GGCACTAGTTTAGTCAACCTCCTCAACGGTGGG
SSB1	YALI0_A00132g	cytosolic heat shock protein of HSP70 family	SSB1f	CGCTCTAGACACAATGAGTGAAGGAACTTTTGCTGGAG
			SSB1r	GGCACTAGTTTATCGGGTAGCCATGGCCTT
PDI1	YALI0_E03036g	disulfide isomerase	PDI1f	CGCTCTAGACACAATGAAGTTCACTGCCCTCACAATTG

			PDI1r	GGCACTAGTTTAAAGCTCATCATCAATCTTGCCTC
LIP2	YALI0_A20350g	Triacylglycerol lipase 2	LIP2f	CGCTCTAGACACAATGAAGCTTTCCACCATCCTTTTC
			LIP2r	GGCACTAGTTTAGATACCACAGACACCCTCGG
LIP2 mRNA	YALI0_A20350g	Triacylglycerol lipase 2	qlip2-r	CTTCAACTGTGGCCTGCAATGT
			qlip2-r	CGATATCTCCTCGGTGGGTGAT
LacZ mRNA	-	β-galactosidase	qlacZ-f	CGATATCTCCTCGGTGGGTGAT
			qlacZ-r	GTGGGCGTATTCGCAAAGGA
HAC1 mRNA	YALI0_B12716g	transcription factor singleton	qhac1-f	CCCACCCTCTTCAACCACGATAATG
			qhac1-r	GGCTGCTTGTTGTCTTCAAACAGGGT
ACT1 mRNA	YALI0_D08272g	Actin	qact1-f	GTCGGCATGGGCCAGAAGGA
			qact1-r	ATGGAGGTGGGGGCAAGAGC

*The restriction enzyme site is single underlined. The homologous sequence for recombination was double underlined.

Table S2 Plasmids used or created in the present study

Plasmids	Description	Source of reference
pYLXP	*URA3*, *LEU2*, *pTEF-tXPR*	[1]
pYL1	*LEU2*, *pTEF-tXPR*	This investigation
pYU1	*URA3*, *pTEF-tXPR*	This investigation
pYL4	*LEU2*, *p4UASTEF-tXPR*	This investigation
pYU4	*URA3*, *p4UATEF-tXPR*	This investigation
pYL8	*LEU2*, *p8UASTEF-tXPR*	This investigation
pYU8	*URA3*, *p8UATEF-tXPR*	This investigation
pYU1-LIP2	*URA3*, *pTEF-LIP2-tXPR*	This investigation
pYU4-LIP2	*URA3*, *p4UATEF-LIP2-tXPR*	This investigation
pYU8-LIP2	*URA3*, *p8UATEF-LIP2-tXPR*	This investigation
pYL8-LIP2	*LEU2*, *p8UATEF-LIP2-tXPR*	This investigation
pYU1-gal	*URA3*, *pTEF-gal-tXPR*	This investigation
pYU4-gal	*URA3*, *p4UATEF-gal-tXPR*	This investigation
pYU8-gal	*URA3*, *p8UATEF-gal-tXPR*	This investigation
pYL8-gal	*LEU2*, *p8UATEF-gal-tXPR*	This investigation
pYL- spliced Hac1 (Kar, Sls, sp14, sp54, Ss5, Ss6, Ss7, Ss8, Sb or Pd)	*LEU2*, *pTEF-* spliced *HAC1* (*KAR2, SLS1*, *SRP14*, S*RP54*, *SSA5*, *SSA6*, *SSA7*, *SSA8*, *SSB1* or *PDI1*)	This investigation
pYU- spliced Hac1 (Kar, Sls, sp14, sp54, Ss5, Ss6, Ss7, Ss8, Sb or Pd)	*URA3*, *pTEF-* spliced *HAC1* (*KAR2, SLS1*, *SRP14*, S*RP54*, *SSA5*, *SSA6*, *SSA7*, *SSA8*, *SSB1* or *PDI1*)	This investigation

Recombinant protein production

The yeast strains were first cultivated in YPD media overnight. And then, this preculture was used to inoculate 100 mL YTD media in a 1 L Erlenmeyer flask at an initial OD value of 0.5. The cultures were incubated at 28°C under continuous shaking at 150 rpm for 5 days. Samples were taken regularly to monitor the growth, enzyme production, glucose consumption and biomass formation.

$Y_{X/s} = \frac{dX}{ds}$ represents the biomass yield coefficient. The biomass and product yields were calculated as the ratio of the amount of biomass or products formed divided by the amount of carbon source consumed. The maximum specific growth rate μ_{max} (h^{-1}) was calculated from the plot of biomass concentration versus time for exponentially growing cells. All the experiments were performed at least in triplicate, and the mean values ± standard deviation were shown.

Table S3 Comparison of the growth of the recombinant *Y. lipolytica* strains expressing Lip2p and β-galp during aerobic cultures on 50 g/L glucose in YTD

a Lip2p production

	YlLIP	Yl4LIP	Yl8LIP	Yl16LIP	Yl16LIP-Hac1	Yl16LIP-Ssa6/Ssa8	Yl16LIP-Ssa6/Ssb1	Yl16LIP-Ssa6/Sls1	Yl16LIP-Ssa6/Pdi1
*μ_{max} (h^{-1})	0.42±0.02	0.41±0.01	0.40±0.02	0.36±0.03	0.39±0.01	0.34±0.02	0.37±0.03	0.35±0.04	0.38±0.02
Yx/s (g DCW·g^{-1})	1.06±0.05	0.99±0.03	1.00±0.03	0.92±0.04	0.98±0.02	0.96±0.03	0.93±0.06	0.95±0.04	0.97±0.05

	Yl16LIP-Ssa8/Ssb1	Yl16LIP-Ssa8/Sls1	Yl16LIP-Ssa8/Pdi1	Yl16LIP-Ssb1/Sls1	Yl16LIP-Ssb1/Pdi1	Yl16LIP-Sls1/Pdi1	Yl16LIP-Ssa6/Ssa8/Ssb1	Yl16LIP-Ssa6/Ssa8/Sls1	Yl16LIP-Ssa6/Ssa8/Pdi1	Yl16LIP-Ssa8/Ssb1/Sls1	Yl16LIP-Ssa8/Ssb1/Sls1/Hac1
μ_{max} (h^{-1})	0.36±0.01	0.37±0.02	0.38±0.02	0.37±0.01	0.36±0.02	0.36±0.03	0.31±0.01	0.33±0.02	0.32±0.03	0.29±0.04	0.38±0.04
Yx/s (g DCW·g^{-1})	0.95±0.03	0.90±0.03	0.86±0.05	0.89±0.03	0.92±0.03	0.94±0.04	0.80±0.02	0.85±0.03	0.89±0.06	0.80±0.05	0.94±0.04

b β-galp production

	Yl-Control (Wildtype)	YlGAL	Yl4GAL	Yl8GAL	Yl16GAL	Yl16GAL-Hac1	Yl16GAL-Ssa5/Ssa6	Yl16GAL-Ssa6/Ssb1	Yl16GAL-Ssa6/Sls1	Yl16GAL-Ssa5/Ssb1
μ_{max} (h^{-1})	0.43±0.01	0.42±0.02	0.41±0.01	0.42±0.04	0.37±0.03	0.37±0.01	0.34±0.02	0.36±0.05	0.35±0.03	0.34±0.03
Yx/s (g DCW·g^{-1})	1.09±0.04	1.04±0.03	0.98±0.04	1.00±0.03	0.99±0.04	0.82±0.06	0.81±0.05	0.79±0.06	0.84±0.07	0.85±0.07

μ_{max}, maximum specific growth rate.

	Yl16GAL-Ssa5/Sls1	Yl16GAL-Ssb1/Sls1	Yl16GAL-Ssa6/Ssa2/Ssb1	Yl16GAL-Ssa5/Ssa6/Sls1	Yl16GAL-Ssa6/Ssb1/Sls1	Yl16GAL-Ssa5/Ssa6/Ssb1/Sls1	Yl16GAL-Ssa6/Ssb1/Sls1/Hac1
μ_{max} (h^{-1})	0.33±0.02	0.34±0.01	0.28±0.02	0.30±0.03	0.25±0.04	0.23±0.03	0.36±0.02
Yx/s (g DCW·g^{-1})	0.88±0.05	0.81±0.04	0.79±0.04	0.74±0.05	0.79±0.06	0.71±0.04	0.91±0.03

The results were calculated from at least three biological replicates, and are given as the mean values ± standard deviation.
